# The Main *Aeromonas* Pathogenic Factors

**DOI:** 10.5402/2012/256261

**Published:** 2012-09-04

**Authors:** J. M. Tomás

**Affiliations:** Departamento Microbiología, Universidad de Barcelona, Diagonal 643, 08071 Barcelona, Spain

## Abstract

The members of the *Aeromonas* genus are ubiquitous, water-borne bacteria. They have been isolated from marine waters, rivers, lakes, swamps, sediments, chlorine water, water distribution systems, drinking water and residual waters; different types of food, such as meat, fish, seafood, vegetables, and processed foods. *Aeromonas* strains are predominantly pathogenic to poikilothermic animals, and the mesophilic strains are emerging as important pathogens in humans, causing a variety of extraintestinal and systemic infections as well as gastrointestinal infections. The most commonly described disease caused by *Aeromonas* is the gastroenteritis; however, no adequate animal model is available to reproduce this illness caused by *Aeromonas*. The main pathogenic factors associated with *Aeromonas* are: surface polysaccharides (capsule, lipopolysaccharide, and glucan), S-layers, iron-binding systems, exotoxins and extracellular enzymes, secretion systems, fimbriae and other nonfilamentous adhesins, motility and flagella.

## 1. Introduction

Ever since the first reference of an organism that could be considered a motile aeromonad in 1891 the taxonomy of the genus *Aeromonas*, initiated in 1943, is complex and continuously changing. Although historically the genus *Aeromonas* was included in the family Vibrionaceae, together with the genera *Vibrio*, *Photobacterium,* and *Plesiomonas*, phylogenetic investigations indicated that they should form their own family: *Aeromonadaceae* [1]. The family Aeromonadaceae consists of Gram-negative, facultative anaerobic, chemoorganotroph bacteria with an optimal growing temperature of about 22°C to 28°C. Generally they are motile by polar flagellation, able to reduce nitrates to nitrites and able to catabolize glucose and several carbohydrates while producing acids and often gases as well. Initially, in *Bergey's Manual of Systematic Bacteriology* this family only included the genus *Aeromonas* and was divided into two principal subgroups: the nonmotile and psycnrophilic species (*A. salmonicida*) and the motile and mesophilic species (*A. hydrophila*, *A. caviae*, and *A. sobria*) [2]. The current edition, list three genera in this family: *Aeromonas*, *Oceanimonas,* and *Tolumonas* [3].

The first classifications within the *Aeromonas* genus have been determined phenotypically (phenospecies), based on growth characteristics and biochemical tests. Nevertheless, there is a great difficulty in identifying the different *Aeromonas* strains on a species level by these characteristics, due to the phenotypical heterogeneity and growing number of known species [4]. One of the biggest steps forward in the taxonomic process was the introduction and continuous use of genotypical methods (genospecies). DNA-hybridisation groups (HG) have been established, associated with the already described phenotypical species. Though some genospecies stay without an associated phenospecies, some phenospecies without associated genospecies and major problems occurred due to differences between the phenotypical and genotypical groups. 

A number of molecular chronometers have been used to evaluate phylogenetic relationships and relatedness among *Aeromonas *species. The ribosomal gene 16S (small subunit) was very useful to classify the Aeromonads [5], and a fast and low-cost method based on Restriction Fragment Length Polymorphisms (RFLP) of the 16S rDNA amplified by polymerase-chain-reaction (PCR) was developed [6, 7]. To better estimate the nucleotide substitution of the 16S-rDNA gene, recent sequence analysis of housekeeping genes, like *gyrB* (B subunit DNA-gyrase) and *rpoD* (*σ* ^70^ factor), has been proposed [8, 9].

According to the latest edition of Bergey's Manual of Systematic Bacteriology [3], 17 species have been officially accepted within the genus *Aeromonas* (DNA-hybridisation groups are indicated in parenthesis): *A. hydrophila* (HG1), *A. bestiarum* (HG2), *A. salmonicida* (HG3), *A. caviae* (HG4), *A. media *(HG5), *A. eucrenophila* (HG6), *A. sobria* (HG7), *A. *veronii [(bv. sobria (HG8) and bv. veronii (HG10)], *A. jandaei* (HG9), *A. schubertii* (HG12), *A. trota* (HG14), *A. allosaccharophila* (HG15) [6], *A. encheleia* (HG16) [10], and *A. popoffii* (HG17) [11]; recently three new species have been described: *A. culicicola* [12], *A. simiae* [13], and *A. molluscorum* [14]. Two DNA-hybridisation groups, *Aeromonas sp.* (HG11) and *Aeromonas* Group 501 (HG13; previously enteric group 501), remain without association with an actual species [15]. 

The members of the *Aeromonas* genus are ubiquitous, water-borne bacteria. They have been isolated from marine waters, rivers, lakes, swamps, sediments, chlorine water, water distribution systems, drinking water, and residual waters, especially during hot months in greater numbers [16]. The number of isolates from drinking water is generally low compared to its numbers found in food. *Aeromonas* strains have been found in different types of food, such as meat, fish, seafood, vegetables and processed foods. Potentially they could represent a serious problem in food, as many strains are able to grow at temperatures of a common refrigerator, at a pH of 4–10 and in presence of higher concentrations of salts [17]. Furthermore it has been shown that they are able to produce exotoxins at low temperatures [18]. 

*Aeromonas* strains are predominantly pathogenic to poikilothermic animals including amphibians, fish and reptiles, whereas they also can be found associated with infections of birds and mammals. In fish, they cause hemorrhagic septicemia that often leads to an elevated mortality and major economic losses in aquaculture. The psicrophilic *A. salmonicida* is considered an important pathogen among a variety of fishes, provoking systemic furunculosis in Salmonidae [19]. Mesophilic species (*A. hydrophila* and *A. veronii*) cause a similar assortment of diseases in fishes as carp, tilapia, perch, catfish, and salmon [20], and *A. hydrophila* and *A. jandaei *provoke aeromoniasis in eels [21]. On the other hand, *A. hydrophila *has been linked to major die-offs and fish kills around the globe over the past decade. 

The mesophilic Aeromonads are emerging as important pathogens in humans, causing a variety of extraintestinal and systemic infections, as well as gastrointestinal infections. Approximately 85% of the clinical isolates of the genus *Aeromonas* consist of two species and a single biotype of a third species: *A. hydrophila*, *A. caviae*, and *A. veronii *sv. sobria [19]. Caused extraintestinal infections include: septicemia, provoked by the dissemination of the organism, from the intestinal tract to systems of circulation, and in the majority observed in immunocompromised patients; wound infections, mostly superficial cutaneous infections, but also infections of tendons, muscles, and bones; infections of the respiratory tract, from epiglottitis to pneumonia; and, less frequent, meningitis, peritonitis, eye infections and hemolytic uremic syndrome [22]. The most commonly described disease caused by *Aeromonas* is the gastroenteritis that can appear in the form of a selflimiting liquid diarrhea to a more severe and invasive diarrhea, which specially is a problem for young children and infants. In the last couple of years also cases of travelling diarrhea caused by *Aeromonas* have been documented [23]. Also, an increased isolation rate of *Aeromonas *species was reported in the floodwater samples following Hurricane Katrina in New Orleans [24], suggesting that this microbe could pose potential public health threats during natural disasters. Despite the demonstration of the enterotoxic potential of some *Aeromonas *strains, there is still a debate on its consideration as an etiological agent, as there were no big epidemical outbreaks described and no adequate animal model is available to reproduce the gastroenteritis caused by *Aeromonas*.

Microbiological infections implicate interaction of host and pathogen. Microbes use their own strategies for survival and multiplication, fighting the defense mechanisms of the host's immune system. The observed clinical manifestations of *Aeromonas* infections suggest that there could be a complex network of pathogenic mechanisms forming of a multifactorial process. Recent studies seem to strengthen this hypothesis as the virulence of this genus depends on the bacterial strain, the infection route, and the animal used as model organism [25]. 

Over the last years there has been a big increase in the number of sequenced genomes of different bacteria in the databases. This information permits a better understanding of the bacteria's potential, though always within limits. To date five complete genomes of genus *Aeromonas* have been sequenced entirely, three of them made available to the public in publications: the strain A449 of *Aeromonas salmonicida*, subspecies *salmonicida* [26], the strain ATCC 7966T of *A. hydrophila* [27], and the strain B565 of *A. veronii* [28]. This information is of great value, although there is a great diversity within the genus and some virulence factors will probably not be present in these strains or these strains show different mechanisms to infect the host than others.

The main pathogenic factors associated with *Aeromona*s are surface polysaccharides (capsule, lipopolysaccharide, and glucan), S-layers, iron-binding systems, exotoxins and extracellular enzymes, secretion systems, fimbriae and other nonfilamentous adhesins, motility and flagella.

## 2. Surface Polysaccharides

### 2.1. Capsule

The capsule (CPS) is a structure composed of polysaccharides that usually covers the outer membrane of the bacterial cell. It is highly hydrated (approximately 95% is water) and made up by repetitions of monosaccharides that are linked within each other by glycosidic bonds that can cause homo- or hetero-polymers. The variety of capsule-forming monosaccharides, different linkage and possible modifications contribute to an additional elevated diversity and structural complexity [29]. This structure frequently forms the most outer layer of the bacterial cell and therefore participates in the bacteria interactions with the environment. In consequence, capsules have been described as a major virulence factor of many pathogens, as they prevent phagocytosis, favor interactions to other bacteria and host tissue, and act as a barrier against hydrophobic toxins [30]. 

*A. salmonicida*, *in vivo* and in TSA medium (Tryptic Soy Agar), is able to form a capsular polysaccharide which is not detectable when growing in liquid TSB medium (Tryptic Soy Broth) [31]. The reported O-chain polysaccharide of *A. salmonicida* produced in TSB and consisting of L-rhamnose, D-mannosamine and D-glucose [32] which was also detected in the bacterial inoculums TSB culture used to prepare the *in vivo* growth chambers. It could be established that the structure of the CPS and lipopolysaccharide (LPS) O-chain polysaccharide of *A. salmonicida* strain 80204-1 produced under *in vitro* growth conditions on TSA. Both polysaccharides were shown by composition, methylation analysis, NMR and MS methods to be composed of linear trisaccharide repeating units containing 3-linked2-acetamido-2-deoxy-D-quinovose, 4-linked 3-[(N-acetyl-L-alanyl)amido]-3-deoxy-D-quinovose and 2-acetamido-2-deoxy-D-galacturonic acid. It has been confirmed by direct different chemical analysis that both CPS and O-chain polysaccharide were also present in the *in vivo*-grown cells of *A. salmonicida* strain 80204-1 harvested at 72 h after implant surgery. These polysaccharides were not detected in the *in vitro*-grown bacterial inoculum TSB culture used for the implants. The role of this structure as a virulence factor was demonstrated, as it reduces opsonization by hindering phagocytosis [33–35] and contributes to the invasion in cell lines of fish [36]. Formation of capsular polysaccharide (CPS) covering the A-layer has been reported to be produced during the *in vivo* culture of *A. salmonicida* in surgically implanted intraperitoneal culture chambers [34]. Moreover, Merino et al. [36] have reported that when grown under conditions promoting capsule formation, strains of *A. salmonicida* exhibited significantly higher ability to invade fish cell lines. It suggests that, as with the A-layer and LPS, CPS is an important virulence factor, essential for host cell invasion and bacterial survival.

Mesophilic *Aeromonas *spp., *A. hydrophila *AH-3 (serogroup O: 34), and *A. veronii *bv. *sobria *(serogroup O: 11) are also able to produce a capsule when grown in glucose-rich media [37]. The strains PPD134/91 and JCM3980 of *A. hydrophila *(serogroup O: 18) also produce capsular polysaccharides, and it was the strain PPD134/91 where genes for biosynthesis and export of the capsule have been described for the first time within the genus. The genetic organization in three regions is similar to the group II of capsular polysaccharides in other bacteria like *Escherichia coli* [38, 39].

### 2.2. Lipopolysaccharide (LPS)

The cell envelope of some Gram-negative bacteria display a form of subcellular differentiation in which peptidoglycan and outer membrane proteins at the cell poles remain stable for generations while material in the lateral walls is diluted by growth and turnover. The outer membrane has a defined *in vivo* organization in which a subfraction of proteins and lipopolysaccharide (LPS) are embedded in stable domains at the poles and along one or more helical ribbons that span the length of the Gram-negative rod [40].

LPS is a surface glycoconjugate unique to Gram-negative bacteria and a key elicitor of innate immune responses, ranging from local inflammation to disseminated sepsis. Gram-negative bacteria have two membrane layers separated by a periplasmic space: an inner or plasma membrane and the outer membrane. LPS is a major component of the outer leaflet of the outer membrane [41] and consists of lipid A, core oligosaccharide (OS), and O-specific polysaccharide or O antigen [41, 42]. The O antigen, which is the most surface-exposed LPS moiety, mediates pathogenicity by protecting infecting bacteria from serum complement killing and phagocytosis [42–45]. O antigens are polymers of OS repeating units. The chemical composition, structure, and antigenicity of the O antigens vary widely among Gram-negative bacteria, giving rise to a large number of O-serogroups [6]. LPS biosynthesis involves a large number of enzymes and assembly proteins encoded by more than 40 genes, recently reviewed in references [46–48]. It begins at the cytosolic or inner membrane, followed by the transit of the molecule to the outer leaflet of the outer membrane where it becomes surface-exposed. The O antigen is synthesized as a lipid-linked glycan intermediate by a process that is remarkably similar to the biogenesis of lipid linked OSs for protein N-glycosylation [49]. The lipid carrier in bacteria is undecaprenyl phosphate (Und-P), while eukaryotic cells and *Archaea *utilize dolichyl phosphate (Dol-P).

The *A. hydrophila* AH-3 WecP represents a new class of UDP-HexNAc: polyprenol-P HexNAc-1-P transferases (ref WecP). These transferase-catalyzed reactions involve a membrane-associated polyprenol phosphate acceptor and a cytoplasmic UDP-D-*N*-acetylhexosamine sugar nucleotide as the donor substrate. Four subgroups of bacterial enzymes have been identified based on their specific substrate preference [50]. *A. hydrophila* AH-3 WecP transfers GalNAc to Und-P and is unable to transfer GlcNAc to the same enzyme substrate [51]. Furthermore, the WecP enzyme (UDP-GalNAc: polyprenol-P GalNAc-1-P transferase) differs from WecA (UDP-GlcNAc: polyprenol-P GlcNAc-1-P transferase) in membrane topology (ref WecP). The differences in substrate specificity and membrane topology between WecP and WecA indicate a different phylogenetical branch [51].

The lipid A is a highly conserved structure and covalently linked to the polysaccharide complex. It is the lipid component of LPS and contains the hydrophobic, membrane-anchoring region of LPS. Lipid A consists of a phosphorylated N-acetylglucosamine (NAG) dimer with 6 or 7 saturated fatty acids (FAs) attached. Some FAs are attached directly to the NAG dimer and others are esterified to the 3-hydroxy fatty acids that are characteristically present. Its biological activity appears to depend on a peculiar conformation that is determined by the glucosamine disaccharide, the PO_4_ groups, the acyl chains, and also the 3-*deoxy*-D-*manno*-octulosonic acid (Kdo) containing inner core. 

The most prominent activity of LPS is its immunostimulatory potency leading to the complex clinical syndrome of Gram-negative sepsis when the initial host response to an infection becomes deregulated. The clinical manifestation of sepsis is characterized by fever, hypotension, respiratory and renal failure, and intravascular disseminated coagulation [52]. These effects are not the result of LPS toxicity, but are rather a consequence of cell activation by LPS and a subsequent deregulation of the inflammatory host response. The biological activity of LPS is harbored in the lipid anchor of the molecule, termed lipid A or “the endotoxic principle” of LPS [53]. The endotoxic LPS properties derive from the release of lipid A of lysed bacteria which can provoke a major systemic inflammation known as septic or endotoxic shock.

The lipid A components of *Aeromonas salmonicida* subsp. *salmonicida* contained three major lipid A molecules differing in acylation patterns corresponding to tetra-, penta- and hexa-acylated lipid A species and comprising 4′-monophosphorylated *β*-2-amino-2-deoxy-D-glucopyranose-(1→6)-2-amino-2-deoxy-D-glucopyranose disaccharide, where the reducing end 2-amino-2-deoxy-D-glucose was present primarily in the *α*-pyranose form [54]. The tetra-acylated lipid A structure containing 3-(dodecanoyloxy)tetradecanoic acid at N-2′,3-hydroxytetradecanoic acid at N-2 and 3-hydroxytetradecanoic acid at O-3, respectively, was found. The penta-acylated lipid A molecule had a similar fatty acid distribution pattern and, additionally, carried 3-hydroxytetradecanoic acid at O-3′. In the hexaacylated lipid A structure, 3-hydroxytetradecanoic acid at O-3′ was esterified with a secondary 9-hexadecenoic acid. Interestingly, lipid A of the *in vivo* rough isolate contained predominantly tetra- and pentaacylated lipid A species suggesting that the presence of the hexa-acylated lipid A was associated with the smooth-form LPS [54].

The core can be subdivided into two regions, the inner and the outer core, based on their sugar composition. The inner core is attached to the lipid A at the 6′ position of one NAG, and all known inner cores contain Kdo or a derivative residue (3-*glycero*-D-*talo*-octulosonic acid). In addition, the base structure of the inner core typically contains L-*glycero*-D-*manno*heptose (L,D-Hep), but some bacteria contain D-*glycero*-D-*manno*heptose (D,D-Hep) alone or in combination with L-D-Hep, while other lack heptoses entirely, like *Rhizobium*. Within a genus or family, the structure of the inner core tends to be highly conserved. In contrast, the outer core provides an attachment site to the O polysaccharide and shows more structural diversity, although variation within a given species, or even a genus, is still limited. The complete lipid A-core OS unit is translocated to the periplasmic face of the inner membrane by the MsbA transporter, which is a member of the glyco ATP-binding cassette (ABC) transporters superfamily requiring ATP hydrolysis [55]. 

The chemical structure of the LPS core of *A. hydrophila* O: 34 [56] and *A. salmonicida* [57]. The complete genomics and proteomics of the LPS core biosynthesis in *A. hydrophila* AH-3 (serogroup O34) were achieved by the identification and characterization of three genomic regions [58]. Combining these data together with the structure elucidation of the LPS core in mutants in each gene from the three gene clusters enabled a presumptive assignment of all LPS core biosynthesis gene functions.

The comparison between the LPS core structures of *A. salmonicida* subsp. *salmonicida* A450 and *A. hydrophila* AH-3 renders a great similarity in the inner and part of the outer LPS core, but some differences in the distal part of the outer LPS core. The three genomic regions encoding LPS core biosynthetic genes in *A. salmonicida* A450 were fully sequenced, being regions 2 and 3 with identical genes to *A. hydrophila* AH-3. *A. salmonicida* A450 region 1 showed seven genes, three of them identical to *A. hydrophila* AH-3, three of them similar but not identical to *A. hydrophila* AH-3 and one of them without any homology to any well characterized gene. Combining the gene sequence and complementation test data with the structural data and phenotypic characterization of mutants, the complete genomics and proteomics of *A. salmonicida *were established [59]. By hybridization studies with internal probes of the *A. salmonicida* specific genes using different *A. salmonicida* strains (besides their subspecies or being atypical), was shown a unique or prevalent LPS core type. 

The O polysaccharide (O antigen) is usually attached to a terminal residue of the outer core and consists of repeating oligosaccharide subunits made up of 1 to 6 sugars. The individual chains vary in length ranging up to 40 repeat units, which constitute the hydrophilic domain of the LPS molecule, as well as a major antigenic determinant of the Gram-negative cell wall. The structural diversity of O-polysaccharides repeated units with more than 60 monosaccharides, different position and stereochemistry of the *O-*glycosidic linkage and presence or absence of different noncarbohydrate substituents least to great variability between species and even strains of Gram-negative bacteria [60]. The variability of O-polysaccharides repeated units, particularly the terminal sugar, confer immunological specificity of the O-antigen. The first useful scheme for the serogroup of *Aeromonas* strains included 44 serogroups based on O antigens for a total of 307 *A. hydrophila* and *A. caviae* strains [61]. Afterwards it was extended to 97 O serogroups [62]. More than 60% of the septicemia cases are related to four of these serogroups: (O: 11; O: 16; O: 18; O: 34) [22]. Serogroup O: 11 is associated with severe infections in humans, like septicemia, meningitis and peritonitis while serogroup O: 34, the most common in mesophilic *Aeromonas*, is associated with wound infections in humans and outbreaks of septicemia in fishes [63]. Furthermore, the LPS of serogroups O: 13, O: 33, O: 34 and O: 44 shows thermoadaptation. Thus, high growth temperatures (37°C) increase the levels of hydroxylated and saturated fatty acids in the lipid A of serogroup O: 34 [64] and in serogroups O: 13, O: 33, O: 34, and O: 44, the S forms of LPS predominate in growth conditions of 20°C or 37°C at higher osmolarity, while R forms predominate at 37°C at lower osmolarity [65, 66].

The chemical structure of *A. hydrophila* O: 34 [67] and *A. salmonicida* subsp. *salmonicida *[68] has been characterized, also the chemical structure of the O: 11 antigen of *A. hydrophila* LL1 with S-layer [69] and *Aeromonas caviae* ATCC15468 [70]. Furthermore, the O antigen biosynthesis genes in the *A. hydrophila* strain PPD134/91 (serogroup O: 18) and AH-3 (serogroup O: 34) have been described [38, 71]. Like in other polysaccharides biosynthesis clusters, three classes of genes have been found: genes involved in the biosynthesis of activated sugars, genes that encode glycosyltransferases, and genes whose products are necessary for the O antigen translocation and polymerization.

The interaction of LPS with cells of the innate immune system leads to the formation and release of endogenous mediators initiating inflammatory and immune responses essential for an antibacterial defense [72]. This primarily protective mechanism may become overshadowed by an acute pathophysiological response with the typical clinical symptoms of septic shock that frequently follows the release of inflammatory mediators, such as tumor necrosis factor (TNF)-a during infection [73]. LPS induces no degranulation in macrophages, but like allergens, it stimulates the de novo synthesis and release of cytokines in these cells. Activation of cells by LPS is mediated by the Toll-like receptor 4 (TLR4), a member of the highly conserved protein family of TLR, which is specialized in the recognition of microbial components. In mice, defects in TLR4 result in LPS unresponsiveness [72]. For functional interaction with LPS, TLR4 requires association with myeloid differentiation protein 2 (MD-2) [74]. According to current consensus activation of TLR4 is preceded by the transfer of LPS to membrane-bound or soluble CD14 by LPS-binding protein (LBP) [75]. This mechanism is believed to be true for LPS signaling generally. However, in a recent study showed that R-form LPS and lipid A, but not S-form LPS, are capable of inducing TNF-*α* responses also in the absence of CD14 [76].

LPSs from *Aeromonas* are mainly high heterogeneous mixtures of S-form LPS molecules containing 1 to over 50 repeating oligosaccharide units and contain ubiquitously a varying proportion of R-form molecules lacking the O-specific chain. Many clinically relevant Gram-negative bacteria synthesize this type of LPS. LPSs are amphipathic molecules whose hydrophobicity decreases with increasing length of the sugar part [77]. Based upon these differences, S- and R-form LPS show marked differences in the kinetics of their blood clearance and cellular uptake as well as in the ability to induce oxidative burst in human granulocytes [78] and to activate the host complement system [79]. In relation to the *Aeromonas* ssp biological activities, like in other Gram-negative bacteria, the lipid A induce the B cell polyclonal activation and the response to immunoglobulin M, both by a T mitogen-independent mechanism. Furthermore, different effects were observed after injection into animals: pyrogenicity, leucopenia followed by leucocytosis, septic shock, hemorrhagic necrosis of tumors, diarrhoea, and also death [80, 81]. On the other hand, the S form of LPS protects the bacteria from the bactericide effects of the nonimmune serum, since the complement component C3b binds to the long O antigen chains being far away from the membrane and unable to form the complement attack complex, and therefore avoids cell lysis [82]. The long O: 34 antigen chains increase hemolytic activity, virulence in fishes and mice [64], and adherence to human epithelial cells [65] and can be considered an important *in vivo *colonization factor [66].

### 2.3. Surface *α*-Glucan

Bacteria, such as *Escherichia coli,* could show up to six distinct saccharide polymers simultaneously present within the glycocalyx. At present, the known components of the saccharide matrix include LPS O-antigens [46], enterobacterial common antigen (ECA) [83], capsular polysaccharides (K-antigen) [84], colanic acid (CA or M-antigen) [85], poly *β*-1,6-*N*-acetyl-D-glucosamine (PNAG) [86], and the *β*-1,4-glucan bacterial cellulose [87]. The *A. hydrophila* AH-3 *α*-glucan (D-Glucose linked *α*1-4 and sometimes branched in a *α*1-6) is a surface polysaccharide exported via the WecP which is also used by the O34-antigen LPS, and ligated to the surface through the O34-antigen polysaccharide ligase (WaaL) [88]. Nevertheless, it is an independent polysaccharide versus the O34-antigen LPS despite the common use of the export and ligation system, because we could find mutants devoid of either polysaccharide or both. The surface glucan is common to the mesophilic *Aeromonas* strains tested. Aeromonas surface glucan production may not have a significant role in cell adhesion but clearly has a role in biofilm formation [88]. Some *E. coli* exopolysaccharides (in particular CA and PNAG [89]) are integral components of biofilms, acting as the “cement,” which holds together the various protein, lipid, and polysaccharide components [90]. A similar role seems to be played *Aeromonas *surface *α*-glucan polysaccharide.

 Several published reports indicate that the use of *β*-glucans enhances *Aeromonas* disease resistance in fish by potentiating innate immunity [91, 92]. The *β*-glucans used are from yeast representing a heterogeneous group of glucose polymers, consisting of a backbone of *β*-(1→3)-linked *β*-d-glucopyranosyl units with *β*-(1→6)-linked side chains of varying length and distribution. However, in no case the authors were able to show the scientific reason for this *Aeromonas* resistance. The fact that *Aeromonas* produces a surface *α*-glucan may explain these results and also suggests that the use of *α*-glucans instead of *β*-glucans could be more helpful to enhance the fish resistance to *Aeromonas* disease [88].

### 2.4. S-layers

 The S-layer is a surface protein layer of paracrystalline nature that is produced by a broad range of bacteria to form the outermost cell envelope. Chemical analysis showed that it is composed of a single protein or glycoprotein (40-200 kDa) and exhibits either oblique, square or hexagonal lattice symmetry with unit cell dimensions in the range of 3 to 30 nm. S-layers are generally 5 to 10 nm thick and show pores of identical size (diameter, 2–8 nm) and morphology. S-layers have been associated with a number of possible functions that relate to pathogenicity. Due to its exposition on the cell surface, it plays a mayor role in diverse biological functions: adhesion, protection against complement and attack by phagocytes, antigenic properties, anchoring site for hydrolytic exoenzymes, bacteriophage receptor, and others [93].

In 1981, Kay and coworkers identified a layer associated with virulence, initially called A-layer, outside the cell wall of *A. salmonicida* [94]. Later, the constituting protein and the sequence of the encoding gene, *vapA*, were identified [95], and it was observed that this layer got lost after growth at temperatures above 25°C, due to a deletion of the genetic material [96]. In parallel, the S-layers of mesophilic *Aeromonas* belonging to serogroup O: 11, and the encoding gene *ahsA *were identified [97]. Although these layers are similar to the one identified in *A. salmonicida* at a morphological level, they differ on the genetic and functional level and could therefore carry out a different role in pathogenicity [98]. More recently the presence of an S-layer was also described in pathogenic isolates of *A. hydrophila* belonging to serogroups O.14 and O: 81 [99]. The S-layer of *Aeromonas *is composed by autoassembling subunits of a single protein that form a tetragonal complex that covers the entire bacterial cell and constitutes the predominant surface antigen [100]. The secretion of *Aeromonas* S-layer subunits involves the cleavage of a signal peptide to be translocated across the plasma membrane, as well as different specific proteins, homologous to components of the type II secretion system (T2SS), to be transferred from the periplasm to the exterior.

The S-layer of *A. salmonicida* promotes association with extracellular matrix proteins and macrophages, binding to porphyrins [101] and immunoglobulins [102], and protection against proteases [100] and oxidative killing [34]. Its presence in the mesophilic *Aeromonas* spp. of serogroup O: 11 increases their capacity of adherence which contributes to the colonization of intestinal mucosa as well as generating a major resistance to opsonophagocytosis which could facilitate systemic dissemination after invasion through the gastrointestinal mucosa [94]. 

Since the early days of S-layer glycoprotein research, it was evident that these cell surface components occur on *Archaea* as well as on bacteria [103]. S-layer glycoproteins have been known for their occurrence among the major lineages of *Archaea*. Among bacteria, for a long time only Gram-positive members of the *Bacillaceae* family have been known to possess S-layer glycoproteins [104]. Only very recently there were the first reports on the occurrence of glycosylated S-layer proteins in the Gram-negative species. Evidence was obtained from biochemical analyses and so far nothing is known about either glycan structure or linkage of the glycans to the S-layer protein portion. However, in contrast to the known S-layer glycoproteins from *Bacillaceae* investigated these glycosylated S-layer proteins originate from potential pathogens and, therefore, might be of medical relevance [105]. Until today all S-layer *Aeromonas *strains have one thing in common: a lipopolysaccharide (LPS) that contains O-antigen polysaccharides of *homogeneous chain* length. This fact leads to the speculation of the possible implication of the LPS in linking the S-layer to the bacterial cell surface [100, 106], or to participate in S-layer glycosylation.

## 3. Iron-Binding Systems

Numerous environments contain less than 1 *μ*M of iron, which is considered the optimum for microbial growth. The low availability of free iron makes bacterial growth and pathogenicity more difficult, but not impossible. Microorganisms developed a series of mechanisms to sequester iron from their hosts or from insoluble polymers of the environment, including reduction of ferric to ferrous iron, occupation of intracellular niches, utilization of host iron compounds, and production of siderophores. While direct evidence that high-affinity mechanisms for iron acquisition function as bacterial virulence determinants has been provided in only a small number of cases, it is likely that many if not all such systems play a central role in the pathogenesis of infection [107]. The competition for iron between the host and the bacterial invader shows the advance of an invasion. Due to the presence of iron-binding proteins in the host, such as hemoglobin, transferrin, lactoferrin, or ferritin, the iron is little accessible *in vivo*. Iron concentrations in the serum are far from the required minimum for growth during the infections of many bacteria. This capacity to deprive an essential nutrient of a microorganism is known as nutritional immunity [108].

Two high affinity mechanisms to acquire iron are known in *Aeromonas* strains: siderophore-dependent and siderophore-independent mechanisms [109]. Siderophores are low-molecular-weight peptides which present functional groups with elevated affinity and specificity towards iron ions. These peptides need specific cell membrane bound receptors as well as a cell-associated apparatus to incorporate the metal into the bacterial metabolism. Mesophilic *Aeromonas* synthesize, enterobactin or amonabactin siderophores, but never both of them. The enterobactin is found in different Gram-negative bacteria, while the amonabactin is only known in *Aeromonas* ssp. Both siderophores are catecholates (phenolates), as they have 2,3-dihydroxybenzoic-acid (2,3-DHB) conjugated with aminoacids [110]. Therefore, their biosynthesis in *Aeromonas* spp. is encoded by two distinct gene groups: the *amo* genes, in strains that produce amonabactin, and the *aeb* genes (aeromonad enterobactin biosynthesis), in enterobactin hydroxypyroridone producing strains [111]. Furthermore, the amonabactin receptor of *A. hydrophila* shows low specificity, permitting the transport of an extraordinary ample range of siderophores, with various chelating groups like catecholate, hidroxamate and hydroxypyroridone [108]. Recently, the* A. salmonicida* gene cluster involved in the catechol-type siderophore biosynthesis has been described [112]. Using a proteomic approach, a recent study demonstrated that under iron-limited conditions *A. salmonicida* expresses three iron-regulated outer membrane receptors, and one of these receptors was proposed to be a putative heme receptor based on sequence homology [113]. Heme uptake in Gram negative bacteria usually involve outer membrane receptors as well as a TonB-dependent internalization process with two accessory proteins ExbB and ExbD, and this system is believed to transduce the energy of the proton motive force of the cytoplasmic membrane into transport energy required by the receptor. Subsequently, transport of heme across the cytoplasmic membrane is driven by ATP hydrolysis, and an ATP-binding cassette (ABC) transporter is involved in this transport. *A. salmonicida* siderophore-independent mechanisms consist of bacterial outer membrane proteins able to bind specific host iron- or heme-binding proteins without the intervention of siderophores [108, 114]. 

The acquisition of iron is recognized as one of the key steps in the survival of bacterial pathogens within their hosts, and contributes significantly to virulence [115]. The expression of genes involved in iron acquisition is tightly regulated by the ferric uptake regulator protein Fur, which acts as an iron-responsive DNA binding repressor [116]. A genetic screening known as the Fur titration assay on this bacterium identify Fur-regulated genes for siderophore biosynthesis and for ferrisiderophore transport previously described. A screening of gene distribution demonstrated that all the analyzed strains shared genes for siderophore biosynthesis and transport and for heme utilization, indicating that these two systems of iron acquisition are a conserved trait [112]. Iron-regulated *A. salmonicida* proteins have demonstrated to be protective antigens for fish and are good candidates for the improvement of vaccines [117].

## 4. Exotoxins and Other Extracellular Enzymes 

### 4.1. Exotoxins

It has been described that the genus *Aeromonas* produces a wide range of exotoxins. However, all toxins described are not produced by all strains, although strains may possess their genes. Furthermore, some strains only express toxin genes in certain growth conditions. Two main types of enterotoxins have been described in *Aeromonas spp*. cytotoxic and cytotonics. 

Cytotoxic enterotoxins, also known as cytolytic enterotoxins, provoke degeneration of crypts and villi of the small intestine and their producing strains are generally isolated from patients suffering diarrhea. These toxins can lead to produce hemolysis, cytotoxicity and enterotoxicity [118, 119]. They are synthesized as a preprotein containing a signal peptide which separates after crossing the inner membrane. The secreted protein is inactive and can be activated by proteolytic nicking near the C-terminus. The active toxin binds to a glycoprotein on the surface of the target cell and oligomerizes forming pores in the host's cell membrane that cause cell death. The cytotoxic enterotoxin Act, from *A. hydrophila *SSU [120], plays an important role in *Aeromonas* infections [121], since it induces early cell signaling in eukaryotic cells, which leads to the production of inflammation mediators in macrophages and in human epithelial cells. Furthermore, it also contributes to apoptosis [122]. Detailed structural-functional studies of the Act enterotoxin and two aerolysins from *A. trota* and *A. bestiarum *[123] showed that these proteins are closely related. However, some heterogeneity at the aminoacid level in some regions could lead to possible differences in folding of these molecules, resulting in differential neutralization of these toxins by specific monoclonal antibodies [124].

Act is an aerolysin-related pore-forming toxin that is responsible for the hemolytic, cytotoxic, and enterotoxic activities of *A. hydrophila*, being its main virulence factor. Hemolysis involves pore formation in the membrane of the target cell and water entry from the external media, resulting in swelling of the cells and subsequent lysis. The toxin interacts with the membranes of erythrocytes, inserts into the lipid bilayer as oligomers, and creates pores in the range of 1.14 to 2.8 nm. Cholesterol serves as the receptor for Act, and the 3′-OH group of this membrane constituent is important for the interaction. Once Act has interacted with cholesterol on the cell membranes, the toxin is activated with subsequent oligomerization and pore formation [125, 126]. The toxin activity also includes tissue damage and high fluid secretion in intestinal epithelial cells, resulting from the induction of a proinflammatory response in the target cells. Act upregulates the production of proinflammatory cytokines such as tumor necrosis factor-alpha (TNF-*α*), interleukin-1 beta (IL-1*β*) and IL-6 in macrophages. TNF-*α* and IL-1*β* stimulate the production of the inducible nitric oxide synthase (iNOS) that, through nitric oxide (NO) production, is an essential element of antimicrobial immunity and host-induced tissue damage. Simultaneously, Act has the ability to activate arachidonic acid (AA) metabolism in macrophages that leads to the production of eicosanoids (e.g., prostaglandin E2 [PGE2]) coupled to cyclooxygenase-2 (COX-2) pathway. AA is a substrate for PGE2 production, but is present at limited concentrations in cells [127]. Act increases the amount of AA from phospholipids by inducing group V secretory phospholipase A2 (sPLA2), which acts in the membrane of eukaryotic cells. Act increases cyclic AMP (cAMP) production in macrophages by indirect activation of adenylate cyclase by PGE2. The *A. hydrophyla* toxin also induces the production of antiapoptotic protein Bcl-2 in macrophages, preventing the occurrence of massive apoptosis resulting from the induction of the inflammatory response, which would be undesirable for the bacteria. Act also promotes an increased translocation of the nuclear factor *k*B (NF-*k*B) and cAMP-responsive element binding protein (CREB) to the nucleus [127]. Transcription factor NF-*k*B is important in a number of inflammation-related pathways. The enhancer/promoter regions of some immunoregulatory cytokine genes, including the TNF-*α*, IL-1*β*, and IL-6, present binding elements for NF-*k*B and CREB [128]. These transcription factors have also important regulatory functions in the transcription of cox-2 and are implicated in the induction of Act cytotoxic activities. The mature protein is 52 kDa and contains 493 aminoacids. It is secreted as an inactive precursor and undergoes processing at both the N- and C-terminal ends to demonstrate biological activity. It has a leader sequence of 23 amino acids that allows the protein to transverse the inner membrane. This leader peptide is removed when the toxin enters the periplasmic space [129].

Cytotonic enterotoxins do not produce degeneration of the epithelium and have mechanisms of action similar to those of the choleric toxin, since they increase the cyclic adenosine monophosphate (cAMP) levels and prostaglandins in intestinal epithelial cells. *Aeromonas* species produces cytotonic enterotoxins that show different molecular weights and variable reactivity to the choleric antitoxin [130, 131]. These enterotoxins have been divided into two groups: heat-labile (56°C for 10 min.), without cross-reactivity with the choleric anti-toxin, and heat-stable (100°C for 30 min.) that react with the choleric antitoxin Chopra and colleagues purified a heat-labile cytotoxic enterotoxin, Alt, from *A. hydrophila* SSU [119] that increased the cAMP levels and prostaglandins in rats intestinal mucosa. The protein sequence shows similarity to the C-terminus of *A. hydrophila* phospholipase C (PLC). They also detected a heat-stable (56°C for 20 min.) cytotonic enterotoxin, Ast, that provokes fluid secretion in rats small intestine and increases the cAMP levels in mucosal cells [119]. 

In addition to cytotoxic hemolytic enterotoxins, *Aeromonas *spp. strains produce at least two other classes of hemolysins without enterotoxic properties: *α*-hemolysins and *β*-hemolysins. The *α*-hemolysins are synthesized in the stationary growth phase and lead to reversible cytotoxic effects and incomplete erythrocytes lysis [132]. The *β*-hemolysins, on the other hand, are usually synthesized in the exponential growth phase. They are thermostable (5 min. at 56°C) and pore forming toxins which lead to osmotic lysis and complete destruction of erythrocytes [17, 132].

### 4.2. Other Extracellular Enzymes

*Aeromonas spp. *secretes a wide range of extracellular enzymes, including proteases, lipases, amylases, chitinases, nucleases, and gelatinases. Although in many cases their role in pathogenicity is still to be determined, they represent a big potential to adapt to environmental changes. Extracellular proteases contribute to the metabolic versatility that allows* Aeromonas *to persist in different habitats and that facilitate ecological interactions with other organism. In general, proteases can contribute to the pathogenicity promoting invasion by direct damage of host tissue or by proteolytic activation of toxins [17]. Furthermore, they can also contribute to the establishment of infection overcoming the initial host defenses, for example, inactivating the complement system, or to providing nutrients for cell proliferation [133]. In *Aeromonas* spp. three different types of proteases have been identified: a temperature-labile serine-protease and two metalloproteases, both temperature-stable but EDTA (ethylenediaminetetraacetic acid)-sensitive and -insensitive, respectively. Additionally, aminopeptidases with a number of specific activities have been described: catabolism of exogenously supplied peptides, extracellular activation of aerolysine, or cleavage of amino-terminal methionine from newly synthesized peptide chains (methionin aminopeptidase) [134].

Lipases or triacylglycerol hydrolases are produced by a wide range of bacteria. They may provide nutrients and constitute virulence factors by interacting with human leukocytes or by affecting several immune systems functions through free fatty acids generated by lipolytic activity. In *A. hydrophila* different lipases, such as the Ah65 lipase/acyltransferase, H3, Apl1 and Lip, have been described [134, 135], with the Apl1 lipase showing phospholipase C activity. In *Aeromonas *spp.serogroup O: 34 two lipases have been described: phospholipase A1 and C. The phospholipase C shows lecithinase and cytotoxic activities and its role as a virulence factor has been demonstrated [136]. Furthermore, glycerophospholipid-cholesterol acyltransferases (GCAT), which digest erythrocytes membranes and leads their lysis, have been isolated from *A. hydrophila *and *A. salmonicida* [134].

## 5. Secretion Systems 

Gram-negative bacteria have a cytoplasmic membrane, a thin peptidoglycan layer, and an outer membrane containing lipopolysaccharide. There is a space between the cytoplasmic membrane and the outer membrane called the periplasmic space. In order to transport proteins to the cell surface or the extracellular space Gram-negative bacteria had developed different secretion systems: secretion systems type I, II, III, IV, V, and VI [137]. This classification is based on proteins transport across the outer membrane and refers to the molecular nature of the transport machinery and the catalyzed reactions. The mechanisms involved in protein transport across the cytoplasmic membrane, in all the above mentioned secretion systems, can be divided into two main groups: Sec-dependent and Sec-independent [138]. Proteins secreted via the Sec-dependent pathway contain an N-terminal signal peptide and utilize the Sec translocase for transport across the cytoplasmic membrane. The Sec-dependent pathway includes the secretion system type II and V. Sec-independent pathways allow the export from the cytoplasm to the extracellular environment in one step and do not involve periplasmic intermediates. These pathways include secretion systems type I, III, IV, and VI, although type IV can also employ the Sec-dependent pathway. An alternative Sec-independent pathway know as twin arginin translocation system (Tat-system), which recognize proteins containing two “twin”-arginine residues in the signal sequence, is employed to transport already folded proteins across the inner membrane [139].

Type III and VI secretion systems (T3SS and T6SS, resp.) have been documented to play a critical role in the virulence of many Gram-negative bacteria, are often activated upon contact with target cells and deliver their toxin proteins, the so-called effectors, directly into the host cells cytosol. 

### 5.1. Type III Secretion System

The T3SS was first identified in pathogenic strains of *Yersinia* spp. [140], and consist of a complex multicomponent system which transports bacterial proteins, frequently involved in pathogenicity, directly from the bacterial cytoplasm across the inner and outer membrane of the bacterial envelope to either the external medium or directly into the eukaryotic cells. The T3SS contains three different types of proteins: (a) structural components that form needle-like structures, so called injectisomes; (b) secretion substrates, so called effectors; (c) chaperones that assist and protect structural and effector proteins during transport. The injectisome consists of approximately 20 different proteins that assemble to form a needle-like structure with thin and rigid hollow needles that extend from the cell surface and are anchored to the envelope by basal structures resembling flagella basal bodies. This structure is usually induced upon contact with the host cells and allows the translocation of the effectors into the eukaryotic cytosol [141].

The T3SS is independent of the Sec system; however the assembly of the secretion apparatus probably requires the Sec machinery, since several components have the characteristic N-terminal signal sequences [142]. The signal that allows effectors recognition and secretion or translocation into the host cells is unknown, although various theories have been suggested [143]. Regardless the differences of these theories it seems that the region that codes the first 20 aminoacids, either in RNA or peptide form, is essential for the effectors recognition and secretion [142].

Until today the presence of a functioning T3SS has been described in *A. salmonicida* [144] and in *A. hydrophila* strains AH-1, AH-3 and SSU [145–147]. The *Aeromonas* T3SS is similar to the *Yersinia* T3SS [146]. Furthermore, four T3SS-effector proteins have been identified in *A. salmonicida*, AexT, AopP, AopO and AopH [148–150], and one, AexT and AexT-like (or AexU), in the *A. hydrophila* strains AH-3 and SSU, respectively [151, 152]. AexT is a bifunctional toxin, homologous to the also bifunctional effectors ExoT/ExoS of *P. aeruginosa*, showing ADP-ribosyltransferase and GAP (GTPase acting protein) activities. AopP belongs to the YopJ family, a group of T3SS effectors that interferes with signaling pathways of mitogen-activated protein kinases (MAPK) and/or the nuclear factor kappaB (NF-*κ*B). The biological functions of AopO and AopH are unknown; nevertheless they are homologues of the *Y. enterocolitica* effectors AopO and YopH, respectively.

### 5.2. Type VI Secretion System

The T6SS was described by Pukatzki and coworkers and seems to constitute a phage-tail-spike-like injectisome, which again serves the purpose of translocations effectors into host cells [153, 154]. It appears to be highly conserved and can be found in one or more copies in diverse Gram-negative species, such as *V. cholerae, P. aeruginosa*, *Y. pestis, E. coli*, *S. enterica, Agrobacterium tumefaciens*, *Rhizobium leguminosarum*, *Francisella tularensis, Burkholderia spp, *and *Edwardsiella spp*. However, the macromolecular structure of this system has not yet been resolved yet, and it is not known how T6SS machines assemble or deliver effectors. A hallmark of all T6SSs is the presence of Hcp (hemolysin coregulated protein) and VgrG (valine-glycine repeat protein G) proteins in culture supernatants [155]. Neither of these proteins is made with a signal peptide, and they are not proteolytically processed. Furthermore, they show structural similarities to components of viral injection machineries indicating that they do not act as classical secreted effectors but are rather surface exposed structural components that might be released in culture supernatants or into eukaryotic cells. Although necessary for *E. tarda *or *Francisella *pathogenesis, T6SS are required for processes as different as resisting predation in *V. cholerae*, symbiosis in *Rhizobium leguminosarum*, biofilm formation in enteroaggregative *E. coli*, killing of niche competitors in *P. aeruginosa*, *Burkholderia thailandensis*, and *V. cholerae*, and stress sensing in *V. anguillarum* for a recent review [156]. 

Several regulatory mechanisms controlling T6SS gene cluster expression have been identified in recent years: they are regulated at the transcriptional level by alternate sigma factors, two-component systems, or transcriptional factors. Several cases of regulation by quorum sensing have also been reported. Because T6SS gene clusters are often found in pathogenicity islands or have been acquired by horizontal gene transfer, their GC content is sometimes different from the GC content of the core genome, and they are silenced by histone-like proteins. T6SS subunit production is also regulated at the translational level through the action of small regulatory RNA, and several T6SS need to be activated by posttranslational mechanisms [157].

Recently, a functional T6SS has been described in *A. hydrophila* strain SSU [158] and its involvement in virulence has been demonstrated. T6SSs gene clusters are also present in the genome of *A. hydrophila* AH-3 and ATCC7966, but their implication in virulence has not been proven. By bioinformatics approach we identified several *Aeromonas* strains with clusters which possess typical −24/−12 sequences, recognized by the alternate sigma factor 54, which directs the RNA polymerase to these promoters which requires the action of a bacterial enhancer binding protein (bEBP), which binds to *cis*-acting upstream activating sequences. Putative bEBPs are encoded within the T6SS gene clusters possessing *σ* ^54^ boxes. 

The importance of *vasH *(*σ* ^54^ activator) and *vasK *of *A. hydrophila *SSU in the expression of the T6SS gene cluster and the secretion and translocation of T6SS associated effectors proteins and their crucial roles in evoking mouse lethality. A *vasH *isogenic mutant was unable to express and produce known T6SS proteins, such as Hcp and VgrG2/3, and *vasK *isogenic mutant was able to express and translocate Hcp into the host eukaryotic cell but unable to secrete it into the extracellular milieu [158]. The proteomics analysis indicated the existence of VgrG1, with its gene localized outside the T6SS gene cluster. This protein has a COOH-terminal extension containing a vegetative insecticidal protein-2 (VIP-2) domain, known for its actin ADP-ribosylating activity (18). VgrG1 is an important virulence factor of *A. hydrophila *that is secreted and also translocated by the T6SS with actin ADP-ribosylating activity [159].

## 6. Adhesins

The bacterial capacity to adhere and colonize the hosts' mucosa is a critical step in the infection process. Two classes of adhesins which allow bacteria to bind to specific receptors on the eukaryotic cell surface have been described in *Aeromonas*: those associated with filamentous structures and those associated with proteins of the outer membrane [160] or other structures.

### 6.1. Filamentous Adhesins: Fimbriae/Pili

Fimbriae/pili are filamentous structures on the bacterial surface, formed by subunits known as pilin. Although pili are often described as adhesive organelles, they have been implicated in other functions, such as phage binding, DNA transfer, biofilm formation, cell aggregation, host cell invasion, and twitching motility. The pili of Gram-negative bacteria have been placed into four groups based on their assembly pathway: (a) pili assembled by the chaperone-usher pathway; (b) the type IV pili; (c) pili assembled by the nucleation/precipitation pathway; (d) pili assembled by the alternative chaperon-usher pathway (CS1 pili family) [161].

In clinical and environmental isolates of mesophilic *Aeromonas*, two distinct types of fimbriae have been found based on their morphology: short, rigid fimbriae (S/R) that can be found in high numbers on the bacterial cell and long, wavy fimbriae (L/W) that can be found in smaller numbers. The S/R fimbriae have a length of 0,6 to 2 *μ*m and are common epitopes in different analyzed species. They are widely distributed (more than 95% of strains) and able to cause autoaggregation, but not hemagglutination or binding to intestinal cells. Furthermore, they are the predominant type in Aeromonads with elevated pili number [162] and in some clinical strains they can be induced under determined environmental conditions (<22°C, in liquid media). The L/W fimbriae are large, fine (4–7 nm), flexible, and considered hemagglutinins. They are also the predominant type in strains isolated from fish which present a small number of pili (<10 per cell). Aminoacid sequence analysis indicates that they correspond to type IV pili [163], known as important structures for adhesion to epithelial cells and involved in biofilm formation and twitching motility. Two different type IV pili have been described in gastroenteritis-associated *Aeromonas* species: the bundle-forming pili (Bfp) and the type IV pili (Tap) [164]. Bfp pili are involved in adhesion to intestinal cells [165] and exhibit N-terminal sequence homology with the mannose-sensitive hemagglutinin pilus of *V. cholerae*. Tap pili differ from Bfp pili in their N-terminal sequences and molecular weights and exhibit highest homology with the type IV pili of *Pseudomonas* and pathogenic *Neisseria* species [164]. Furthermore, one of the Tap-family proteins (TapD) is essential for secretion of aerolysin and proteases, contributing to type II secretion [163]. Recently, the role of Bfp in *A. veronii *bv. *sobria* adherence, by the study of a 22-kb locus encoding the bundle-forming that contained 17 pilus-related genes similar to the mannose-sensitive hemagglutinin (MSHA) of *V. cholerae*. The bundle-forming pilus is required for *A. veronii *adherence and biofilm formation, and both the major and minor pilin proteins are essential for this process [166].

*A. salmonicida *subsp. *salmonicida*, a bacterial pathogen of Atlantic salmon, has no visible pili, yet its genome contains genes for three type IV pilus systems. One system, Tap, is similar to the *P. aeruginosa *Pil system, and a second, Flp, resembles the *Actinobacillus actinomycetemcomitans *Flp pilus [167]. The Tap pili appeared to be polar, while the Flp pili appeared to be peritrichous. The Tap pilus made a moderate contribution to virulence, while the Flp pilus made little or no contribution. Unlike the pili of other piliated bacterial pathogens, *A. salmonicida *subsp. *salmonicida *type IV pili are not absolutely required for virulence in Atlantic salmon. 

### 6.2. Nonfilamentous Adhesins

On the *Aeromonas* spp. surface there are also other macromolecules considered adhesins, like the S-layer monomers, the lipopolysaccharide and different outer membrane proteins. Among the outer membrane proteins, the porins have been specially described to act like a lectin-type adhesins, binding the bacteria to carbohydrate-rich surfaces like erythrocytes and probably intestinal human cells [168].

## 7. Motility and Flagella

### 7.1. Motility

Depending on the environmental conditions, bacteria are able to move in a free individual manner or remain in the same place to form colony groups and colonize surfaces. Bacteria living in surface colonies have several advantages over single cells. As a group, bacteria can optimize growth and survival by the presence of different cell types that are able to perform specialized functions; can have better access to nutrients; better defense mechanisms for protection against unfavorable environmental conditions such as desiccation. Furthermore, bacteria in colonies secrete polysaccharides to form biofilms which enhance adhesion, survival, and movement.

Different types of bacterial movement have been described: swimming, swarming, gliding, twitching, and sliding among others. All of them are associated to movement over surfaces; except for swimming, that is also used for motility in liquid media. It has been shown that the twitching requires type IV pili, like several forms of gliding, whereas other forms of gliding remain to be explained. The sliding represents a form of passive translocation. Only swimming and swarming are correlated with the presence of flagella. However, whereas swimming is an individual endeavour, swarming is the movement of a group of bacteria. Swimming in liquid media alternates between straight and tumbling movements. In bacteria with peritrichous flagella, such as E. coli, the counter-clockwise (CCW) flagella rotation results in the formation of a helical bundle that propels the cell forward in one direction in a smooth-swimming motion, a so-called run. By contrast, the clockwise (CW) rotation causes unbundling of the helical bundle, allowing the bacterium to randomly reorient its direction, so-called tumbling. In bacteria with a single polar flagellum, such as *Aeromonas*, CCW rotation propels the cell forward in a run, whereas CW rotation propels the cell backward with a concomitant random reorientation. About 60% of mesophilic *Aeromonas *strains showed swarming motility [169].

### 7.2. Flagella 

#### 7.2.1. Structure

Within the Gram-negative bacteria, the most studied model of flagellum has been that of *E. coli* and *S. enterica* sv. Typhimurium [170]. The prokaryotic flagellum has been structurally divided into an external part, constituted by the filament and the hook, and an internal part embedded in the bacterial surface, the so-called basal body. *E. coli* and *Salmonella* express approximately 20.000 copies of a single flagellin (FliC). However, *V. parahaemolyticus* is able to synthesize 6 different polar flagellins [171], *A. caviae* presents two polar and two lateral flagellins, and *A. hydrophila* presents two polar and only one lateral flagellin [172–174].

The flagellar motor is divided into two substructures: the rotor and the stator. The rotor, composed of the FliM, FliN, and FliG proteins that form the C ring structure at the base of the flagella basal body, and the stator, consisting of membrane-embedded proteins surrounding the MS-ring, that constitute proton or sodium ion channels and couple the flow of ions to flagella rotation [175]. In the proton-driven motor of *E. coli* and *S. enterica* serovar Typhimurium, the stator is composed of two integral membrane proteins, MotA and MotB [175] polar flagella stator of *Vibrio* species, such as *V. alginolyticus* and *V. parahaemolyticus*, require four proteins: PomA, PomB, MotX, and MotY [171]. MotP/PomA and MotS/PomB proteins are homologous to the proton-driven MotA and MotB, respectively. MotX and MotY do not have paralogous proteins in *E. coli* and are components of the T-ring [176], which is located beneath the P-ring of the polar flagella basal body in *Vibrio* species.

Mesophilic *Aeromonas* also showed two additional polar stator genes named *pomA*_2_ and *pomB*_2_. *A. hydrophila *PomA_2_ and PomB_2_ are highly homologous to other sodium-conducting polar flagella stator motors. *Aeromonas* PomA-B and PomA_2_-B_2_ are redundant sets of proteins, as neither set on their own is essential for polar flagella motility either in aqueous or high-viscosity environments. Both PomA-B and PomA_2_-B_2_ are sodium-coupled stator complexes although PomA_2_-B_2_ is more sensitive to low sodium concentrations than PomA-B [177]. Furthermore, the stator of the mesophilic *Aeromonas *proton-driven lateral flagella motor (MotA_L_ [LafT] and MotB_L_ [LafU]) is additional to the polar flagella stators [169, 177].

#### 7.2.2. Genetics

**Figure 1:**
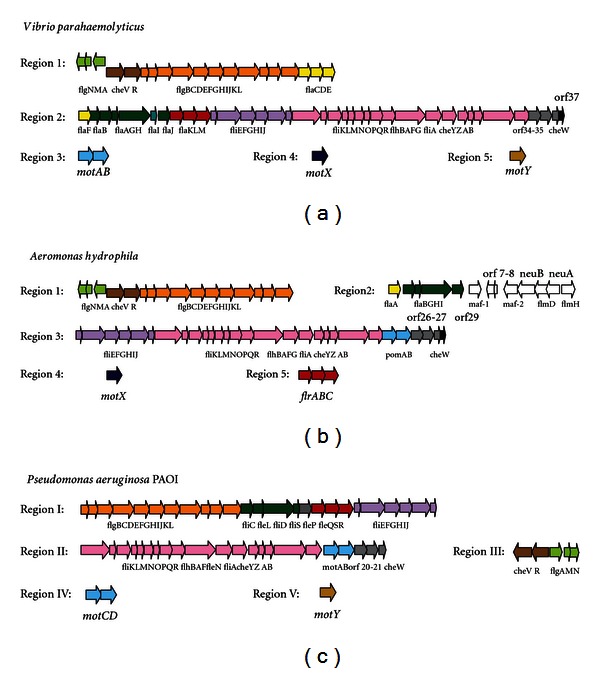
Genetic organization of polar flagellar genes in *V. parahaemolyticus*, *A. hydrophila* and *P. aeruginosa*.

In bacteria with polar flagella as *Vibrio spp.*, mesophilic *Aeromonas* and *Pseudomonas aeruginosa*, genes are distributed in at least five chromosomal regions, but the majority is located in two of them (Figure 1) [171, 174]. 

Region 1 is similar in *Vibrio* and *Aeromonas*, though *Vibrio* possesses three more flagellin gene (*flaCDE*). Organization of the *fla* genes in region 2 is also similar in *Vibrio* and *Areomonas,* with the difference that the *fli* genes of region 2 in *Vibrio* are found in *Aeromonas*' region 3. However, *Vibrio* also possesses one more flagellin gene (*flaF*) and a gene encoding a putative chaperone (*flaI*) between *flaH* and *flaJ*. Furthermore, downstream of the *Aeromonas flaJ* lies a gene which encoded a homolog of the Maf proteins reported in *H. pylori*, *Clostridium acetobutylicum, *and *Campylobacter jejuni *[178]. *Aeromonas* polar flagella region 3 shows similar organization to the genes downstream of *flaM* in *Vibrio *region 2 with the absence of the motor genes. No master regulatory genes encoding homologues of *Vibrio parahaemolyticu*s FlaK, FlaL and FlaM or *P. aeruginosa* FleQ, FleS, and FleR, were found upstream of *A. hydrophila fliE*. In contrast, *Aeromonas* have two genes (*pomA* and *pomB*) which encode orthologues of the MotA and MotB, motor proteins of *Pseudomonas*. Region 4 of *Vibrio* and *Aeromonas* includes a gene that encodes the sodium-driven motor protein MotX which is involved with MotA, MotB and MotY in torque generation of polar flagellum; however in *Pseudomonas* this region contains genes that encode the motor proteins MotCD. Region 5 of *Vibrio* and *Pseudomonas* includes a gene that encodes the motor protein MotY, however in *Aeromonas* it contains the master regulatory genes (*flrABC*). Recently, the *Aeromonas *MotY has been found in a different region with different behavior than the *Vibrio* MotY [179].

**Figure 2:**
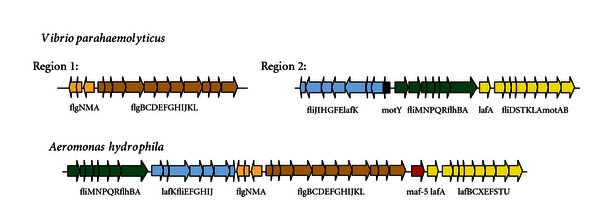
Genetic organization of lateral flagella genes in *V. parahaemolyticus *and *A. *hydrophila [180, 181].

The complete set of genes involved in the formation of a functional and inducible lateral flagella system was described in two bacterial species: *V. parahameolyticus* and *A. hydrophila*. These lateral flagella are encoded by 38 genes distributed in two discontinuous regions (region 1 and 2) on chromosome II in *V. parahameolyticus* [180] and in one unique chromosomal region in *A. hydrophila* [181] (Figure 2). In these two bacterial species, the polar and lateral flagella systems do not share either structural or regulatory genes. A partial set of genes was described in other bacteria with functional dual flagella, such as *A. brasilense*, *A. caviae*, *R centenum,* and *B. japonicum* [169]. 

*V. parahaemolyticus* region 1 genes are divided into two divergent transcribed sets of genes: *flgAMN*_*L*_ and *flgBCDEFGHIJKL*_*L*_. Homologous *A. hydrophila* genes exhibit the same distribution and direction of transcription. *V. parahaemolyticus* region 2 genes are arranged in four clusters*: fliMNPQRLflhBA*_*L*_, *lafA* and *fliDSTKLA*_*L*_ *motAB*_*L*_ transcribed in the same direction, and *motYLlafKfliEFGHIJ*_*L*_ transcribed in the opposite direction. Homologous *A. hydrophila* genes are transcribed in the same direction and do not contain any homologous gene to *V. parahaemolyticus* motY_L_. Furthermore, the *A. hydrophila* lateral flagella region contains, between *flgL*_*L*_ and *lafA*, a modification accessory factor gene [178], *maf5*, which is independently transcribed.

The expression of flagellar genes is highly regulated, due to the energetic cost of biosynthesis and use of the flagellum for the bacteria. Flagellar systems are regulated by numerous environmental factors and global regulators of the bacteria. To ensure the maximum efficiency and precision of the biosynthesis, the bacteria use hierarchic regulatory networks that include transcriptional and posttranslational mechanisms to control expression of the flagellar components. In *E. coli* and *S. enterica* sv. Typhimurium models for peritrichous flagellation a hierarchy of three transcriptional classes were established. The early genes that correspond to the operon *flhDC* are controlled by class I promoters (independent of *σ* ^70^) and code for the transcriptional activator, FlhD_2_C_2_, for the next genes in the hierarchy. The class II genes (also independent of *σ* ^70^) code components of the exportation complex, the basal body, the hook, the flagellum specific sigma-factor *σ* ^28^ (FliA) and the anti-*σ* ^28^ factor (FlgM). FlgM is a negative regulator that binds to *σ* ^28^ and therefore inhibits its function. During flagellum biosynthesis, when the structure of the basal body is completed, FlgM is secreted out of the cell by the flagellum exportation complex, resulting in FlgM-depletion in the cytoplasm and release of *σ* ^28^ to activate the late genes [182]. The late genes (class III) are activated by *σ* ^28^ and include the system of chemotaxis, the motor, HAPs, and the flagellins. 

There have also been described regulatory cascades with four transcriptional classes of bacteria with polar flagellation, like *V. cholerae* and *P. aeruginosa*. The early genes express the protein FlrA in *V. cholerae* and FleQ in *P. aeruginosa*, which binds to the *σ* ^54^ factor and thus activates the class II genes. Class II genes include two-component-regulators, *V. cholerae flrBC* or *P. aeruginosa fleSR*, and *fliA* (*σ* ^28^). FlrC and FleR are regulators that also associated with the *σ* ^54^ factor and activate class III promoters, whereas *σ* ^28^ factor activates class IV genes (figure D) [183]. In *V. parahaemolyticus* possible regulatory cascades for its two flagellar systems have been described. The *V. cholerae* and *P. aeruginosa* model was proposed for regulating the polar flagellum with the regulators *flaK*, an early gene, and *flaLM*, class II genes [180]. On the other hand, the lateral flagellar system is regulated by LafK. This transcriptional regulator, that shows similarity to FlrA and FleQ, associates with the *σ* ^54^ factor to activate the expression of class II genes. Within these genes we find the gene that codes the *σ* ^28^ factor, which activates the late genes [180]. In this species it was also observed that the loss of the FlaK-regulator of polar flagellation can be compensated by LafK, the lateral regulator [184].

*A. hydrophila* polar flagellum class I gene transcription is *σ* ^70^-dependent, which is consistent with the fact that *A. hydrophila* polar flagellum is constitutively expressed [185]. In contrast to other bacteria with dual flagella systems such as *V. parahaemolyticus*, the* A. hydrophila* LafK protein does not compensate for the lack of the polar flagella regulator FlrA (*V*.* parahaemolyticus *FlaK homologue). This is consistent with the fact that the *A. hydrophila* FlrA mutation abolishes polar flagella formation in liquid and on solid surfaces but does not affect inducible lateral flagella formation. The results highlight that the polar and lateral flagella inter-connections and control networks are specific and that there are differences between the dual flagellar systems in *A. hydrophila* and *V. parahaemolyticus*. Furthermore, the *A. hydrophila* polar flagellum transcriptional hierarchy (also in class II, III and IV genes) shares not only some similarities, but also many important differences with those of *V. cholerae *and *P. aeruginosa* [185]. 

**Figure 3:**
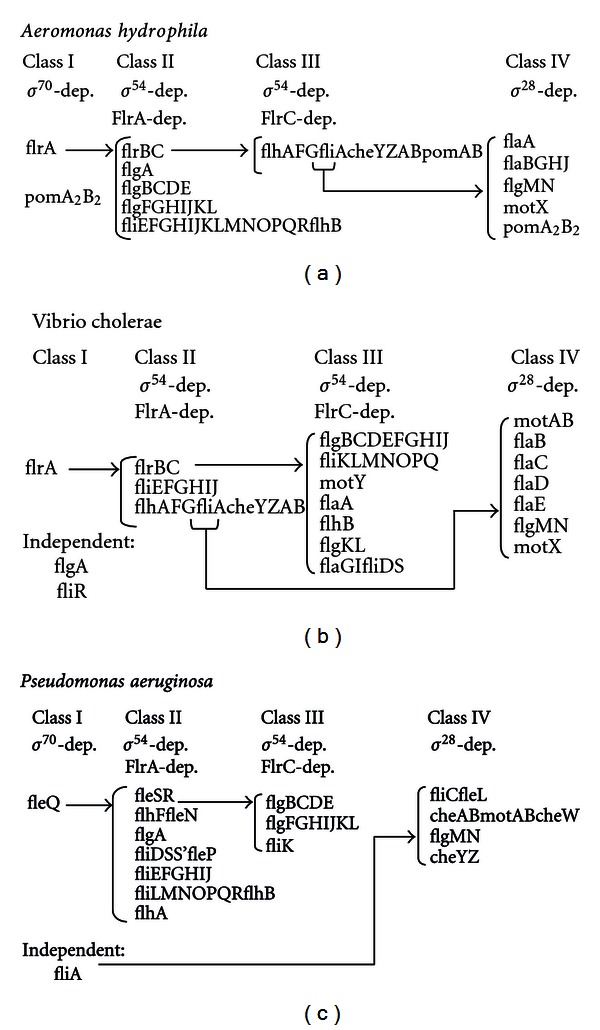
Polar flagellum gene transcription hierarchies.

Comparative proposed *A. hydrophila*,* V. cholerae* [186], and *P. aeruginosa *[183] polar flagellum gene transcription hierarchies are shown in Figure 3.

#### 7.2.3. Virulence Factor

The flagella of pathogenic bacteria promote the colonization and invasion of the host's mucosa. Once the bacteria reach the mucosa the flagellum structure becomes necessary for motility, adhesion and invasion. This motility, coupled with chemotaxis, permits the pathogens to reach the target-tissue of the mucosa. In *Helicobacter pylori *and* P. aeruginosa *motility is crucial for the infection of stomach and lungs, respectively. *V. cholerae *motility is necessary to colonize the intestinal mucosa. In *P. mirabilis* the swarming is associated with provoking important urinary tract infections. Motility is associated with the invasion of epithelial cells in *Y. enterocolitica*. Furthermore the flagella are reported to act like adhesins, most probably via its flagellin dominions D2 and D3 [187]. The flagellin of *P. aeruginosa* is able to bind to the lungs mucine [188]. Some enteropathogenic strains of *E. coli* adhere to the intestinal mucosa via a flagellum-dependent mechanism [189]. Motility and presence of flagella is also associated with biofilm formation, which generally goes along with persistent infections. 

The colonization of the mucosa provokes a proinflamatory response or inducible innate immune response, mainly stimulated by specific cells of the mucosa. In mammals TLR5 (Toll-like receptor 5) is implicated in flagellin recognition by its dominion D1 [190]. TLR5 stimulate the transcription of the NF-*κ*B (nuclear factor-kappaB) and MAPK (mitogen-activated protein kinase) dependent proinflamatory genes. Systemic injection of flagellins in mice induces the production of proinflammatory cytokines like TNF-*α* (Tumor Necrosis Factor *α*), IL-6 (Interleukine 6) and nitric oxide. In epithelial cells flagellins of *E. coli*, *S. enterica* sv. Typhimurium and *P. aeruginosa* stimulate the secretion of IL-8, the essential chemokine to attract neutrophiles and macrophages to the infection site [187]. Additionally, glycosylation of the flagellin protein was shown to play a role in the proinflammatory action of *P. aeruginosa* flagellin. IL-8 release from A549 cells stimulated with nonglycosylated flagellin was significantly reduced when compared to wildtype flagellin [191]. Similar pathogenic mechanisms have been observed for *Aeromonas* polar and lateral flagella ([174], [181], and unpublished results).

#### 7.2.4. Glycosylation

Glycosylation is the most abundant polypeptide chain modification in nature. Glycans can be covalently attached to the amide nitrogen of Asn residues (*N*-glycosylation), to the hydroxyl oxygen of, typically, Ser or Thr residues (*O*-glycosylation), and, in rare cases, to the indole C2 carbon of Trp. Protein glycosylation was first demonstrated in the late 1930s and was long thought to exist only in eukaryotes. As more than two-thirds of eukaryotic proteins are predicted to be glycosylated [192] and these modifications are essential for a multitude of cellular functions [193], it is not surprising that countless publications have been dedicated to this topic. It took 40 years until the first bacterial and archaeal glycoproteins were discovered on the surface layers (S-layers) of the archaeon *Halobacterium salinarum *[194] and on the S-layers of two hyperthermophilic *Clostridium *species [195, 196].

*O*-linked protein glycosylation occurs in all three domains of life, and the eukaryotic and bacterial pathways are well characterized. In this section, we focus on the *O*-glycan pathways that modify bacterial flagella and pili. Our understanding of bacterial flagellin- and pilin-specific *O*-glycosylation systems has also been growing, and general *O*-glycosylation pathways have been identified. A distinguishing feature is that the *O*-linkages can be formed with Ser, Thr or Tyr residues [197].

More than 20 years later, the first bacterial *N*-linked protein glycosylation (Pgl) pathway was described in the bacterium *C. jejuni *[198].Examples of surface-associated glycoproteins in Gram-negative bacteria are the pilins of *P. aeruginosa *and *Neisseria* spp., the adhesins TibA and AIDA-1 of *E. coli*and HMW1of *Haemophilus influenzae, *and the flagellins of *P. aeruginosa*, *H. pylori*, *Clostridium botulinum*, and *C. jejuni*/*C. coli *[199]. These species solely possess a polar flagellar system and the filaments are constituted by the two flagellins FlaA and FlaB. The structural characterization of the flagellins in this species led to identify the presence of pseudaminic acid (Pse5Ac7Ac) decorated in *O*-linkage to serine (Ser) or threonine (Thr) residues in the central region of the primary sequence, which is predicted to be surface exposed in the assembled filament [200]. Pseudaminic acid (5,7-diacetamido-3,5,7,9-tetradeoxy-L-*glycero*-L-*manno*-nonulosonate acid) is a sugar of 9 carbon-atoms, similar to *N*-acetylneuroaminic acid or sialic acid (Neu5Ac). In *C. jejuni* also other residues that modify flagellins have been found, all of them deriving from pseudaminic acid [201], whereas in *C. coli* flagellin modification with pseudaminic acid and two derivates of the related nonulosonate sugar legionaminic acid (5,7-diacetamido-3,5,7,9-tetradeoxy-D-glycero-D-galacto-nonulosonic acid, Leg5Ac7Ac) was reported. Up to 19 glycosylation sites were found in both *Campylobacter* flagellins and these modifications represent about 10% of the total protein mass In *H. pylori*, 7 glycosylation sites in FlaA and 10 in FlaB were found. The glycosylation sites do not seem to be related to a certain conserved peptide sequence, though a hydrophobic region next to the Ser/Thr residues was often described [202].

At the genetic level, glycosylation islands (GI) have been identified in *P. aeruginosa* and *P. syringae*. The GI of *Campylobacter* appears to be one of the most variable loci in the genome, containing in between 25 and 50 genes, depending on the strain, that is situated close to those of the flagellins [203]. A number of genes of this locus encode proteins with homology to carbohydrate biosynthetic enzymes, including some (*neu*-locus) with homology to sialic acid biosynthesis enzymes [204]. Extensive mutational analyses, in addition to novel metabolomic analyses and functional studies on recombinant enzymes, have defined the precise function of a number of GI genes in the Pse5Ac7Ac biosynthetic pathway and provided preliminary evidence for the role of the *ptm* genes from this locus in the legionaminic acid biosynthetic pathway [201]. In addition to carbohydrate biosynthetic genes, the *Campylobacter* GI contains multiple copies of hypothetical genes encoding proteins which belong to the motility accessory factor (MAF, Cj1318 family) of proteins. Insertional inactivation of individual copies of these genes has provided evidence for a role in motility or glycosylation although the precise function of each MAF gene remains to be determined [205].

The functionality of glycosylated flagellins and the precise nature of glycosylation in pathogenic bacteria are still to be determined. In some species, like *C. jejuni* and *H. pylori*, glycosylation is necessary for filament assembly [205]; whereas the absence of glycosylation does not affect assembly nor motility in *P. aeruginosa*. Flagellar glycosylation reportedly plays an important role in intestine colonization of *C. jejuni* [203] and the proinflammatory action of *P. aeruginosa* [191]. In *P. syringae* the flagellum-glycosylating residues determine the specificity to the host plant and also play a role in stabilization of the filament structure [206].

In *A. caviae,* the polar flagellins, FlaA and FlaB, were shown to be glycosylated, at six or seven sites, respectively, with a novel nonulosonate sugar derivate of 373 Da mass, and glycosylation was required for flagellar assembly [207, 208]. Though bacterial adherence to Hep-2 cells requires polar flagella, it is not yet known if the novel glycan plays a role in this process [209]. *A. caviae *Sch3N possesses a small genomic island that is involved in both flagellin glycosylation and LPS O-antigen biosynthesis. This island appears to have been laterally acquired as it is flanked by insertion element-like sequences and has a much lower G-C content than the average aeromonad G-C content. Most of the gene products encoded by the island are orthologues of proteins that have been shown to be involved in pseudaminic acid biosynthesis and flagellin glycosylation. Two of the genes, *lst *and *lsg*, are LPS specific as mutation of them results in the loss of only a band for the LPS O-antigen. The proteins encoded by *flmA*, *flmB*, *neuA*, *flmD*, and *neuB *are thought to make up a pseudaminic acid biosynthetic pathway, and mutation of any of these genes resulted in the loss of motility, flagellar expression, and a band for the LPS O-antigen [210]. Studies on lateral flagella from *A. hydrophila *have indicated that their flagellins are also glycosylated, although the structure of the glycan has yet to be determined [173]. Of significance is the identification of a homologue (*maf5*) of the *Campylobacter* MAF family of genes, in the lateral flagella structural locus of *A. hydrophila* [181]. The product of this gene is required for lateral flagella production and provides the first preliminary evidence that glycosylation may also be required for lateral flagella assembly in this species. 

*A. hydrophila* strain AH-3 in-frame deletion mutants of pseudaminic acid biosynthetic genes *pseB* and *pseF* homologues resulted in abolition of polar and lateral flagella formation by posttranscriptional regulation of the flagellins, which was restored by complementation with wildtype *pseB* or *F* homologues or *Campylobacter pseB *and *F* [211]. Polar and lateral flagellin proteins from *A. hydrophila* strain AH-3 (serogroup O34) were found to be glycosylated with different carbohydrate moieties. The lateral flagellin was modified at three sites in *O*-linkage, with a single monosaccharide of 376 Da, which we show to be a pseudaminic acid derivative. The polar flagellin was modified with a heterogeneous glycan, comprised of a heptasaccharide, linked through the same 376 Da sugar to the protein backbone, also in *O*-linkage [212].
